# Priming by Chemokines Restricts Lateral Mobility of the Adhesion Receptor LFA-1 and Restores Adhesion to ICAM-1 Nano-Aggregates on Human Mature Dendritic Cells

**DOI:** 10.1371/journal.pone.0099589

**Published:** 2014-06-19

**Authors:** Kyra J. E. Borgman, Thomas S. van Zanten, Carlo Manzo, Raquel Cabezón, Alessandra Cambi, Daniel Benítez-Ribas, Maria F. Garcia-Parajo

**Affiliations:** 1 ICFO-Institut de Ciències Fotòniques, Barcelona, Spain; 2 Department of Gastroenterology, Hospital Clinic de Barcelona, IDIBAPS, Barcelona, Spain; 3 Department of Tumor Immunology, Radboud Institute for Molecular Life Sciences, Radboud University Medical Center, Nijmegen, The Netherlands; 4 Centro de Investigación Biomédica en Red de Enfermedades Hepáticas y Digestivas (CIBERehd) and Centre Esther Koplowitz, Barcelona, Spain; 5 ICREA-Institució Catalana de Recerca i Estudis Avançats, Barcelona, Spain; University Hospital Münster, Germany

## Abstract

LFA-1 is a leukocyte specific β_2_ integrin that plays a major role in regulating adhesion and migration of different immune cells. Recent data suggest that LFA-1 on mature dendritic cells (mDCs) may function as a chemokine-inducible anchor during homing of DCs through the afferent lymphatics into the lymph nodes, by transiently switching its molecular conformational state. However, the role of LFA-1 mobility in this process is not yet known, despite that the importance of lateral organization and dynamics for LFA-1-mediated adhesion regulation is broadly recognized. Using single particle tracking approaches we here show that LFA-1 exhibits higher mobility on resting mDCs compared to monocytes. Lymphoid chemokine CCL21 stimulation of the LFA-1 high affinity state on mDCs, led to a significant reduction of mobility and an increase on the fraction of stationary receptors, consistent with re-activation of the receptor. Addition of soluble monomeric ICAM-1 in the presence of CCL21 did not alter the diffusion profile of LFA-1 while soluble ICAM-1 nano-aggregates in the presence of CCL21 further reduced LFA-1 mobility and readily bound to the receptor. Overall, our results emphasize the importance of LFA-1 lateral mobility across the membrane on the regulation of integrin activation and its function as adhesion receptor. Importantly, our data show that chemokines alone are not sufficient to trigger the high affinity state of the integrin based on the strict definition that affinity refers to the adhesion capacity of a single receptor to its ligand in solution. Instead our data indicate that nanoclustering of the receptor, induced by multi-ligand binding, is required to maintain stable cell adhesion once LFA-1 high affinity state is transiently triggered by inside-out signals.

## Introduction

Leukocyte specific integrins are a subfamily of heterodimeric α/β transmembrane receptors involved in adhesion and migration of white blood cells. These receptors allow leukocytes to act upon the detection of potential threats to the body by enabling rapid anchoring of leukocytes to the inner wall of blood vessels. This process is followed by leukocyte migration from the bloodstream to the site of inflammation. In a subsequent step of the immunological cascade, leukocytes stably adhere to other immune cells to communicate the detected threat [1]–[4]. Lymphocyte Function-associated Antigen-1 (LFA-1) is a member of the leukocyte specific integrin family, and belongs to the subgroup of β_2_ integrins. This receptor has been found on the membrane of multiple types of leukocytes [5], including lymphocytes, monocytes, and dendritic cells. The main binding partner of LFA-1 is Intercellular Adhesion Molecule-1 (ICAM-1), which is highly expressed on activated endothelial cells and Antigen Presenting Cells (APCs) such as dendritic cells [6], [7]. LFA-1 facilitates rolling, arrest and transendothelial migration during the extravasation of monocytes and lymphocytes by binding to ICAM-1 on the endothelial cells [8], [9]. The interaction between LFA-1 and ICAM-1 also plays a role in the formation of the immunological synapse between lymphocytes and APCs [10]. To successfully accomplish these different types of adhesion in distinct cell types, tight regulation of LFA-1 activity is crucial.

For a long time, affinity [11]–[13] as well as avidity [14], [15] have been recognized as important factors regulating LFA-1 activity. Affinity refers to the adhesion capacity of a single receptor to its ligand in solution, and it is determined by the molecular conformation of the receptor [11]. High resolution TEM data has shown that LFA-1 can be found in at least three different conformational states [11]: bent down with a low affinity for the ligand, transiently extended with an intermediate affinity for the ligand, and fully extended with a high affinity for the ligand [12]. Avidity, on the other hand, refers to the binding strength of a multitude of receptors together that effectively contribute to adhesion [14]. Affinity of the single molecules defines part of this binding, but the extra component in avidity is determined by the spatial organization of the receptors. Indeed, we have previously shown on resting monocytes that organization of LFA-1 in nanoclusters [15] and in hotspot regions together with lipid rafts [16] contributes to avidity. More recently, we have also shown that lateral diffusion of the receptor across the membrane is crucial in the regulation of LFA-1 activity [17]. Using single molecule approaches, we demonstrated that LFA-1 is primarily mobile on resting monocytes with a small sub-population of stationary nanoclusters [17]. Using conformation-dependent antibodies we identified the small stationary LFA-1 sub-population as consisting of extended activated molecules, while the mobile population is mostly bent down and inactive. Importantly, we found that this small subset of stationary activated LFA-1 molecules (accounting for only 5% of the total LFA-1 population) is sufficient to initiate sites for adhesion, being reinforced by the contribution of mobile low-affinity nanoclusters [17].

During differentiation of monocytes into dendritic cells, LFA-1-mediated binding to ICAM-1 is lost while expression levels of the receptor remain constant [15], [18]. Activating mature DCs (mDCs) with chemokine CCL21 increases the population of extended LFA-1 molecules and also restores LFA-1 adhesive properties [18]. CCL21, also known as SLC, is a chemokine that regulates the homing of lymphocytes and dendritic cells from distant sites to lymphoid tissues [19]–[21] via binding to its receptor CCR7 [22], [23]. It has been shown in lymphocytes that soluble CCL21 triggers the high-affinity conformation of LFA-1 [24] and induces binding of LFA-1 to its ligand ICAM-1 [25]. This functional data on LFA-1 activation by chemokines suggest a dramatic change in the modulation of receptor activity in the process of cell differentiation and chemokine activation, i.e. from a loss to a chemokine-induced transient regain of LFA-1 binding capacity. Considering that LFA-1 conformation state and lateral diffusion on the cell membrane are highly coupled [17], [26], it is conceivable that potential changes on the mobility of the receptor on mDCs after CCL21 stimulation might contribute together with affinity to the regain of LFA-1 functionality. Yet, dynamic studies of LFA-1 mobility on resting and chemokine activated mDCs supporting the functional changes in adhesion and ligand binding [18], [25] are lacking so far. Moreover, how modulation of LFA-1 function during differentiation is achieved and how CCL21 cooperates to regulate LFA-1 activity on mDCs remains unknown. Here we have performed systematic single particle tracking studies to directly report on the lateral mobility of LFA-1 on both monocytes and mDCs. We show that LFA-1 exhibits higher mobility on resting mDCs compared to monocytes. CCL21 stimulation of the high affinity state of LFA-1 on mDCs led to a significant reduction of LFA-1 lateral mobility and an increase on the fraction of stationary receptors, with overall diffusion profiles that closely resemble those obtained on resting monocytes. Addition of soluble monomeric ICAM-1 in the presence of CCL21 did not alter the diffusion profile of LFA-1 while soluble ICAM-1 nano-aggregates in the presence of CCL21 further reduced LFA-1 mobility. We finally shed new light on how Talin1, a cytoplasmic protein known to contribute to integrin function regulation and activation [24], [27]–[31] by binding the β_2_ subunit of LFA-1 to the actin cytoskeleton [32], [33], could be involved in β_2_ integrin activation. Our data shows that Talin1 plays a different role in basal LFA-1 regulation on monocytes and mDCs. Overall, our results underscore two main features associated to LFA-1 activation and function. First, lateral mobility of the receptor is directly correlated with its activation state, with LFA-1 priming resulting in restricted lateral diffusion. Second, chemokines are required but not sufficient to maintain the high affinity state of the receptor, which is stabilized by multi-ligand binding.

## Materials and Methods

### Cell Culture

Mature dendritic cells (mDC) were derived, as reported previously [34], from peripheral blood samples. Buffy coats from healthy donors were obtained from *Banc de Sang I Teixits* upon written informed consent. In brief, peripheral blood mononuclear cells (PBMCs) were allowed to adhere to a plastic surface for 2 h at 37°C. Unbound PBMCs were washed away, and the remaining adherent monocytes were cultured for 5 days in a 37°C, 95% humidity, 5% CO_2_ incubator in the presence of IL-4 (300 U/ml) and GM-CSF (450 U/ml) (both from Miltenyi Biotec, Madrid, Spain) in X-VIVO 15 (BioWhittaker, Lonza Belgium) medium supplemented with 2% AB human serum (Sigma-Aldrich, Spain). After 5 days, DCs were matured for 48 hours using a cocktail of IL-1β, IL-6 (both at 1000 IU/ml), TNF-α (500 IU/ml) (all 3 from CellGenix, Freiburg, Germany) and Prostaglandin E2 (PGE2, 10 µg/ml; Dinoprostona, Pfizer). mDCs were harvested and brought to the proper concentration for subsequent experiments.

Monocytes in solution were positively selected from PBMCs using anti-CD14 magnetic microbeads (Miltenyi Biotec, Madrid, Spain).

### Flow Cytometry

For flow cytometry analysis, mDCs were labeled with primary antibody anti-CCR7 (R&D systems), followed by secondary staining with PE-labelled goat-anti-mouse (from BD Pharmingen), both for 30 min at 4°C and a concentration of 5 µg/ml. Appropriate isotype control IgG1 (from BD Pharmingen), was included. Flow cytometry was performed using FACSCanto II.

### Sample Preparation for Single Dye Tracking (SDT)

Monocytes and mDCs were prepared to track individual LFA-1 molecules on the membrane. Chambered cover glasses (8 wells, Nunc Lab-Tek II) were incubated with Poly-L-Lysine (10 µg/ml, Sigma-Aldrich) for 30 min. Fresh cells were diluted up to a concentration of 5×10^5^ per ml in RPMI, and attached to the bottom of the cover glasses by incubation for 30 min. Unbound cells were washed away, and the sample was blocked for 15 min with 2% of HS in RPMI at 37°C. LFA-1 was then labeled by incubation with TS2/4-Atto647N conjugates in a concentration of 0.01 µg/mL for 3.5 min to allow for single dye tracking experiments, i.e., at sub-labeling conditions. Afterwards, the sample was washed thoroughly and 350 µl of RPMI per well was used as imaging medium. All incubation steps were done at 37°C and washing in between steps was done with RPMI without supplements. During the experiments where mDCs were activated, 50 µl CCL21 (final concentration in experiments 1 µg/ml; Recombinant Human CCL21/6Ckine, R&D systems) was added during the measurements and mobility was measured before and up to 10 min after addition at one-minute intervals. Experiments is which mDCs and monocytes were stimulated with ICAM-1 were designed similarly, using either 1 µg ICAM-1 monomers in 50 µl RPMI (Recombinant Human, R&D systems) (a concentration known to saturate high affinity LFA-1 on lymphocytes [35]), or 50 µl ICAM-1 nano-aggregates (100 µg/ml ICAM-1 incubated for 30 min at 37°C with 100 µg/ml polyclonal anti-ICAM-1 (Santa Cruz) [16]) with a final experimental concentration of 20 µg/ml. In combination experiments, 50 µl RPMI containing a cocktail of ICAM-1 and CCL21 was added to the samples.

### Single Dye Tracking

Freshly prepared samples were placed on the microscope setup, and movies of typically 300 frames with a frame rate of 10 Hz were recorded. We used a home build setup arranged around an inverted microscope (IX70, Olympus) with a 1.45 NA oil immersion objective (PLAPON 60×0TIRFM, Olympus). Samples were illuminated using a 633 nm HeNe laser (circularly polarized light, 1.4 kW/cm^2^) in oblique illumination mode to excite the samples slightly above the glass-cell interface, minimizing in this way any potential artifacts associated with the proximity of the cell membrane to the glass substrate. The fluorescence emission of the ATTO-647N was separated from the excitation light using a dichroic mirror (Semrock, FF500/646-Di01). A 660 nm long pass filter (Semrock, BLP01-635R-25) then selectively allowed the fluorescent emitted light to be detected by an EMCCD (Hamamatsu) camera. Temperature was maintained at 37°C with 5% CO_2_ by a custom made incubator built around the microscope stage. In experiments where cells were stimulated with CCL21 and/or ICAM-1, movies of 30 s were recorded before, and at one-minute intervals after stimulation up to 10 minutes.

### Single Trajectory Analysis

Analysis was done as previously described [17]. In brief, two-dimensional trajectories in the plane of focus were reconstructed based on a colloidal particle-tracking algorithm translated to MatLab. For each individual trajectory longer than 13 frames, a mean square displacement (MSD) curve was generated and the diffusion coefficient was calculated using a linear fitting through the second to the fourth point (D_2–4_). A semi-logarithmic histogram was generated containing the short-time lag (D_2–4_) diffusion coefficients from multiple trajectories in different cells. Since the mobility was rather slow and the cells were extensively stretched, typically around 150 trajectories were recovered per cell. The cut-off value to define the mobile population was calculated by generating a similar histogram on fixed cells, and defining the diffusion coefficient for which 95% of the fixed population had a lower or equal D value. In our experimental conditions, the cut-off value was D≤0.001 µm^2^/s.

We then generated an overall mean square displacement plot of the total mobile population up to a time lag of 2 seconds. A linear fitting through the first 4 points was used to define the average diffusion coefficient of the mobile LFA-1 population. In addition, we also used Cumulative Probability Distribution (CPD) analysis as described earlier by us [17] to separate the mobile LFA-1 population into 2 different subpopulations or fractions: slow and fast. The percentage of each fraction within the total mobile population was estimated, and the diffusion coefficient of each fraction was calculated as described above. Individual data points are the mean value of all trajectories measured per each cell.

### Sample Preparation for ICAM-1 Binding Assay

FluoroDishes (35 mm, World Precision Instruments) were incubated with Poly-L-Lysine (10 µg/ml, Sigma-Aldrich) for 30 min. Fresh cells were diluted up to a concentration of 1×10^6^ per ml in RPMI, and attached to the bottom of the cover glasses by incubation for 30 min. For the last 2 minutes, cells were incubated with either ICAM-1 dimers (20 µg/ml ICAM-1 incubated for 30 min at 37°C with 20 µg/ml monoclonal anti-ICAM-1 (BD Pharmingen)), ICAM-1 dimers plus CCL21 (1 µg/ml), ICAM-1 nano-aggregates (20 µg/ml ICAM-1 incubated for 30 min at 37°C with 20 µg/ml polyclonal anti-ICAM-1 (Santa Cruz)), or ICAM-1 nano-aggregates plus CCL21 (1 µg/ml). Immediately after these 2 minutes, samples were fixed using 2% paraformaldehyde (PFA) for 15 min at RT. Then, cells were blocked for 1 h at RT with 3% BSA 2% HS and 20 mM glycine in PBS, followed by primary labeling for 30 min at RT with 5 µg/ml of either, in the case of the dimers, goat-anti-mouse-ATTO647N or, in the case of the nano-aggregates, goat-anti-rabbit-AF647 (Invitrogen). Finally all samples were fixed again with 2% PFA.

### Imaging ICAM-1 Binding

Imaging was performed using a confocal microscope (TCS SP5, Leica Microsystems). Images were taken with a 1.4 NA oil immersion objective (HCX PL APO CS 63.0x, Leica), a 512×512 pixel format and a scanning speed of 400 Hz. Both dimers labeled with ATTO647N and aggregates labeled with AF647 were excited with the 633 nm line at 35% of the HeNe laser power and detected between 645 nm and 715 nm. Images of individual cells were taken with a line accumulation of 3 times and a frame average of 14 times.

### Sample Preparation for Talin Localization Assay

Polydimethylsiloxane (PDMS) elastomer stamps were fabricated by curing for 1–2 hours by 80°C Sylgard 184 (Dow Corning) in a 10∶1 weight proportion (base: crosslinker), on a silanized silicon patterned master (patterns of 2.5×2.5 µm) generated in photoresist using standard photolithography techniques. The cured PDMS was peeled off the master and cut into 1×1 cm squares. PDMS stamps were sonicated in ethanol for 10 min, rinsed with milliQ water and blow dried with nitrogen gas. Stamps were coated with 5 µg/ml of either mouse IgG1 (Sigma Aldrich), TS2/4 (Biolegend) or 50 µg/ml F(ab)^2^ goat-anti-human (Jackson Immunoresearch), and free Atto647N dye to visualize the location of the positive areas. After 45 min of incubation, stamps were rinsed with milliQ water, blown dry with nitrogen gas, and stamped into clean (sonicated for 10 min. in 1∶1 ethanol: miliQ) and hydrophilic (10 min exposure to UV/Ozone Bioforce nanosciences) glass coverslips (Menzel- Glaser Ø30 mm #1). A circle with a DAKO pen was drawn to keep all subsequently used solutions on the coverslip. After stamping, the negative areas on the glass surface were blocked for 30 min. at 37°C with 3% bovine serum albumin (BSA) in PBS to minimize unspecific binding. The F(ab)^2^ goat-anti-human samples were subsequently incubated for 1 hour at 37° with 5 µg/ml ICAM-1. After washing with PBS, cells were added in a concentration of 1×10^6^ per ml and were allowed to adhere to the substrate for 30 min at 37°C. Part of the samples were activated for the last 2 minutes with CCL21 (1µg/ml) Afterwards, cells (monocytes or mDCs) were rapidly fixed with 2% paraformaldehyde (PFA) for 15 min at RT, and permeabilized for 10 min RT with 0.05% Saponin (Sigma-Aldrich) in PBS with 10 mM glycine to allow intracellular labeling. After blocking for 20 min at RT with PBS 1% BSA, cells were labeled with 9 µg/ml antiTalin-ATTO488 conjugates for 40 min at RT. Finally, samples were fixed again and stored at 4°C until measurement.

### Imaging Localization of Talin and LFA-1

Imaging was performed using a confocal microscope (TCS SP5, Leica Microsystems). Images were taken with a 1.4 NA oil immersion objective (HCX PL APO CS 63.0x, Leica), a 512×512 pixel format and a scanning speed of 400 Hz. AntiTalin-Atto488 was excited with the 488 nm line, at 25% of the argon laser power and detected between 500 nm and 570 nm. Atto647N (positive squares) was excited with the 633 nm line at 4% of the HeNe laser power and detected between 645 nm and 715 nm. Images of individual cells were taken with a line accumulation of 3 times and a frame average of 12 times, focusing on the contact area between cell and substrate.

### Analysis Localization of Talin to LFA-1

We used the fluorescent images of Talin (green) and positively patterned areas (red) to quantify the degree of localization of Talin to LFA-1. We first selected the cell area in both channels, and created from the red channel a mask of the pattern (positive = 1, negative = 0). We applied this mask to the green channel, and calculated the average intensity per area of the green Talin signal in each positive square. To define the degree of enhancement in each positive area, this local average was divided by the average green intensity per area of the entire negative part of the mask covering the cell. To avoid artifacts, we excluded the negative region just around (up to 10 pixels away) a positive region.

### Statistical Analysis

**Figure 1:**
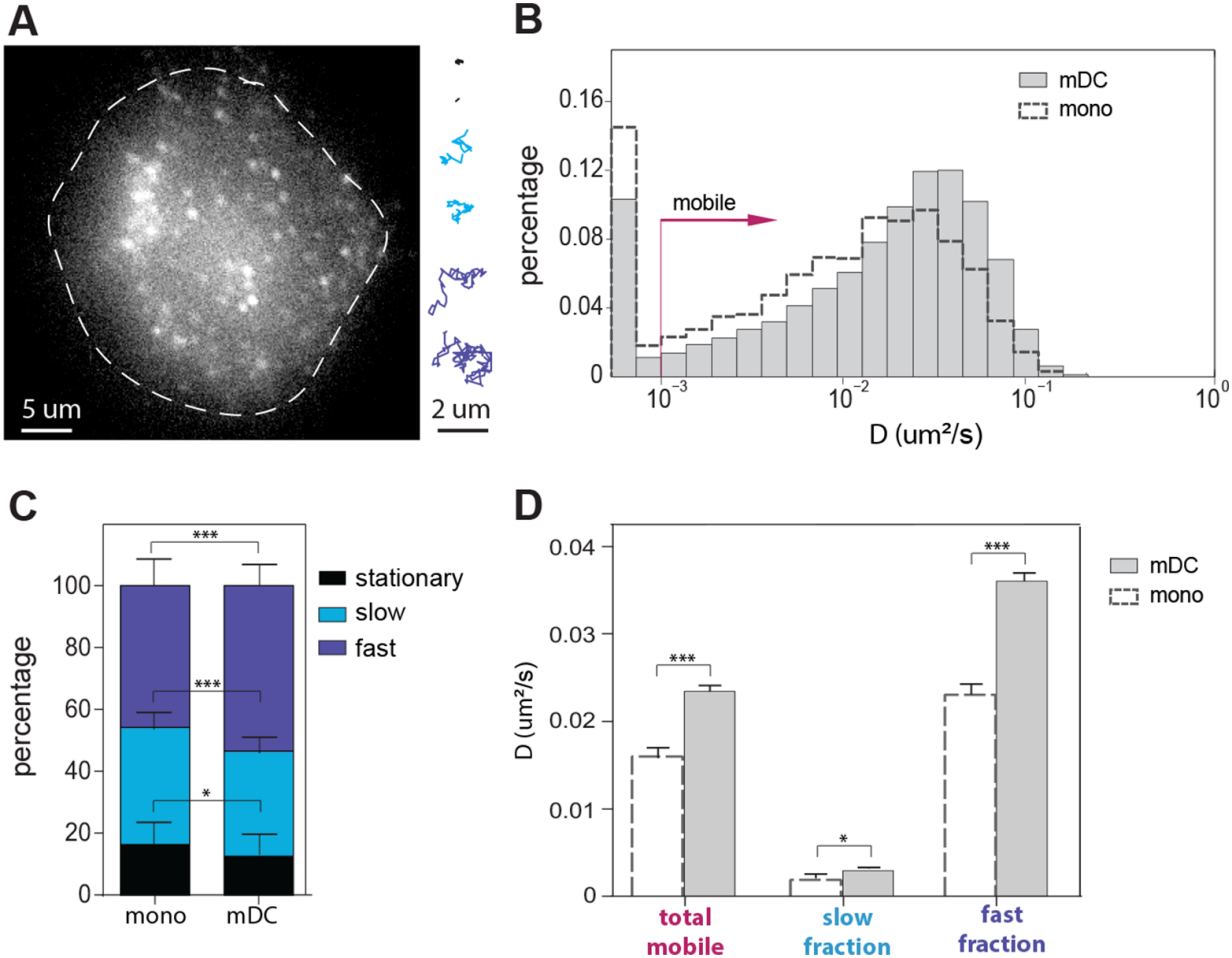
Mobility of LFA-1 on monocytes and mDCs. (**A**) Representative frame from a recorded movie on a mDC to which SDT was applied (see Movie S1). Representative examples of stationary, slow and fast trajectories are displayed. (**B**) Overlay semi-log histogram of LFA-1 diffusion on both monocytes (dashed black lines) and mDCs (grey bars). (**C**) Percentage of stationary, slow and fast diffusing LFA-1 molecules on monocytes and mDCs, as extracted from the cumulative probability distribution analysis (see Methods). (**D**) Diffusion coefficient of the total mobile population, and slow and fast diffusing fractions of LFA-1 on monocytes and mDCs. 25 monocytes divided over 6 independent samples (4587 trajectories) and 117 mDCs from 5 different donors, divided over 117 independent samples (26756 trajectories) were imaged. Means ± SEM are depicted. The Student T-test was used to determine significant differences between means. The resulting P values are indicated as follows: *P<0.05; ***P<0.0001.

**Figure 2:**
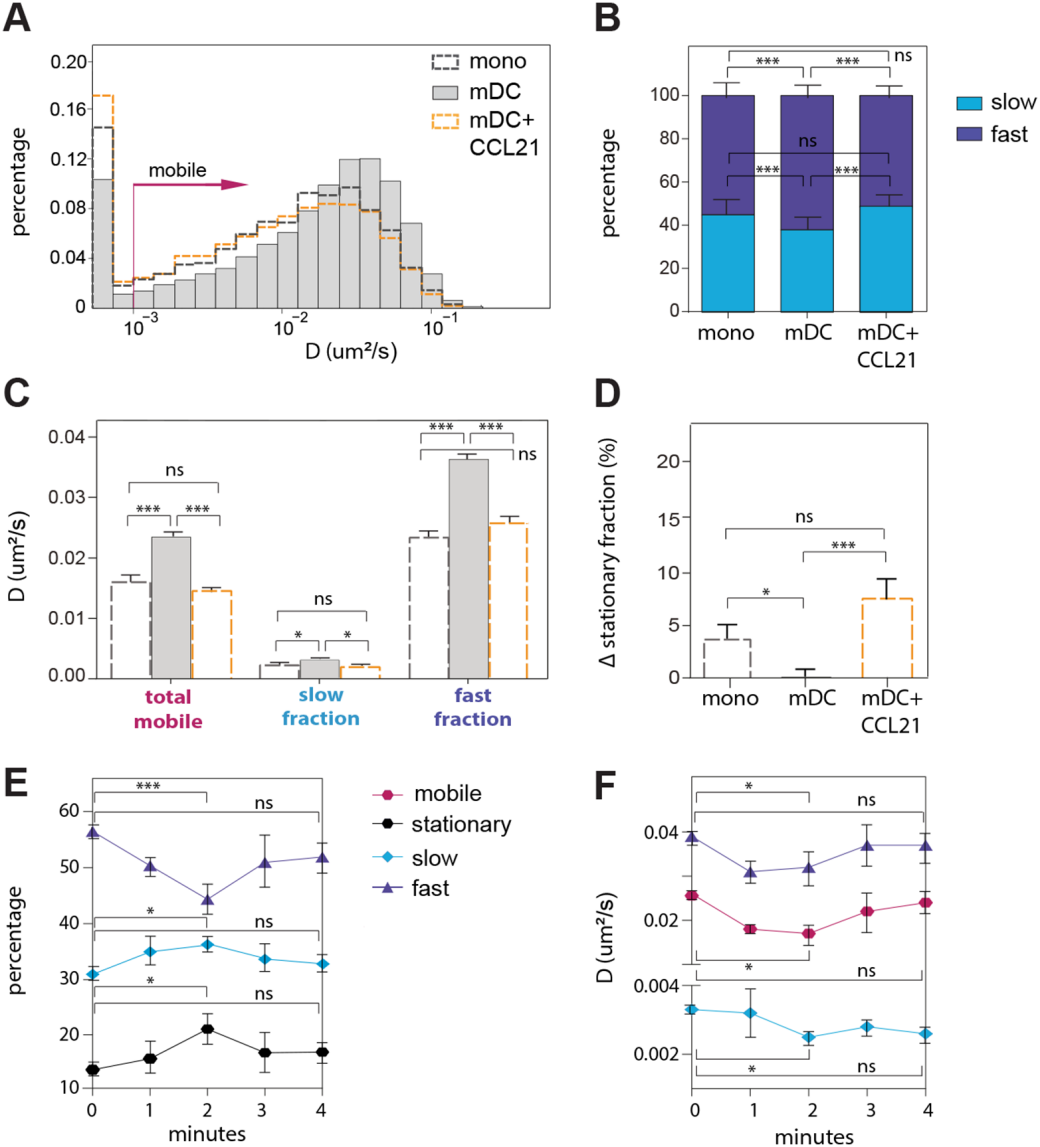
Mobility of LFA-1 on mDCs after activation with CCL21. (**A**) Overlay histograms of LFA-1 diffusion on monocytes (4578 trajectories), resting mDCs (26756 trajectories) and CCL21 activated mDCs (4213 trajectories). (**B**) Percentage of total mobile LFA-1 population (normalized to 100%) displaying slow and fast diffusion on monocytes, mDCs and 2 min CCL21 activated mDCs. (**C**) Diffusion coefficient of the total mobile population, and slow and fast diffusing fractions of LFA-1 on monocytes, mDCs and 2 min CCL21 activated mDCs. (**D**) Stationary fraction of LFA-1 on monocytes, mDCs and CCL21 activated mDCs, displayed as the difference from the total stationary fraction on mDCs, which serve here as the default. Data from **A–D** on CCL21 activated mDCs is based on 22 cells in independent experiments from 3 different donors. (**E**) Percentage of the stationary, slow and fast diffusing LFA-1 molecules at different time points after CCL21 activation. (**F**) D values for the total mobile, and slow and fast fractions of LFA-1 at different time points after CCL21 activation. Data from **E** and **F** is based on 11 cells, 11 independent samples and around 2000 trajectories per time point. **A**–**F** Means ± SEM are depicted. The One-way ANOVA followed by the Tukey multiple comparison test were used to determine significant differences between means. The resulting P values are indicated as follows: *ns (P*>0.05); * (P<0.05) and *** (P<0.0001).

**Figure 3:**
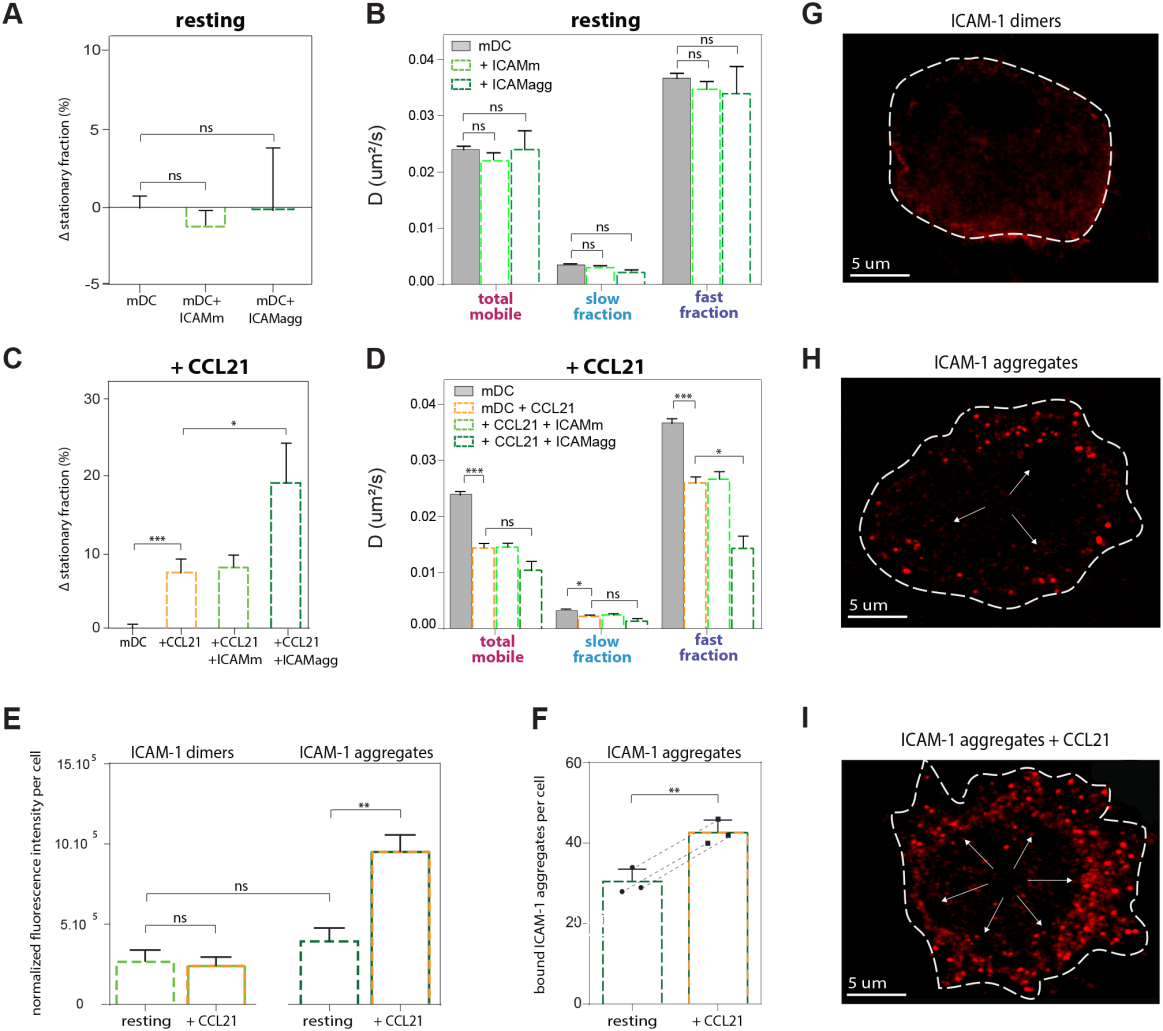
Mobility of LFA-1 on resting and CCL21-activated mDCs after soluble monomeric and nano-clustered ICAM-1. (**A,B**) ICAM-1 (either monomeric: ICAMm, or as nano-aggregates: ICAMagg) was added to resting mDCs and mobility was measured before, and between 1 and 5 min after addition. (**A**) Stationary fraction of LFA-1 molecules on resting mDCs and mDCs + either monomeric ICAM-1 or ICAM-1 nano-aggregates, displayed as the difference with respect to the total stationary fraction on resting mDCs, which serve here as the default. (**B**) Diffusion coefficient of the total mobile population, and slow and fast diffusing fractions of LFA-1 on resting mDCs and after addition of either monomeric ICAM-1 or ICAM-1 nano-aggregates. 30 cells divided over 2 independent experiments (3393 trajectories) were imaged for the ICAMm condition and 10 cells (684 trajectories) for ICAMagg. (**C, D**) ICAM-1 (either monomeric or nano-aggregates) was added together with CCL21 to mDCs and mobility was measured before, and 2 minutes after addition. (**C**) Stationary fraction of LFA-1 molecules on resting mDCs (serving as reference control), CCL21 activated mDCs and CCL21 activated mDCs + either monomeric ICAM-1 or ICAM-1 nano-aggregates, displayed as the difference with respect to the total stationary fraction on resting mDCs. (**D**) Diffusion coefficient of the total mobile population, and slow and fast diffusing fractions of LFA-1 on resting mDCs, CCL21 activated mDCs and after simultaneous addition of CCL21 and either monomeric ICAM-1 or ICAM-1 nano-aggregates. 16 cells (8423 trajectories) from 2 different donors divided over 5 independent samples (ICAMm) and 7 cells (314 trajectories) from 2 different donors divided over 7 independent samples (ICAMagg) were imaged. (**E**) Quantification of fluorescent ICAM-1 dimers (monomers bound together due to antibody labelling) and nano-aggregates binding in resting and CCL21 activated mDCs, normalized to the area quantified and to the background signal outside of the cell. For this, regions of the cell in between the obvious fluorescent ICAM-1 aggregates were selected, the fluorescent intensity was measured using ImageJ, and used to compare the baseline fluorescent signal across all 4 conditions. 20 cells per condition were imaged. (**A–E**) Means ± SEM are depicted. The One-way ANOVA followed by the Tukey multiple comparison test were used to determine significant differences between means. The resulting P values are indicated as follows: *ns (*P>0.05); * (P<0.05), ** (P<0.001) and *** (P<0.0001). (**F**) Quantification of bound ICAM-1 nano-aggregates to resting and CCL21 activated mDCs. After applying a threshold of 25% of the fluorescent signal, all visible fluorescent spots per cell were counted. 20 cells per donor and 3 different donors were imaged. Each data point represents the mean value for 1 donor. Means ± SEM and individual data points are depicted, and dotted lines connecting datapoint of the same experiment indicate that not just in average, but in each individual experiment using a different donor, an increase of ICAM-1 nano-aggregate binding is observed after CCL21 activation. The paired two-tailed Student T-test was used to determine significant differences between means. (**G–I**) Representative examples of confocal images of ICAM-1 binding to mDCs: (**G**) dimeric ICAM-1 to resting cells, (**H**) nano-aggregates of ICAM-1 to resting cells and (**I**) nano-aggregates to CCL21 activated cells. Arrows in **H** and **I** point to the binding of individual ICAM-1 nano-aggregates to LFA-1.

**Figure 4:**
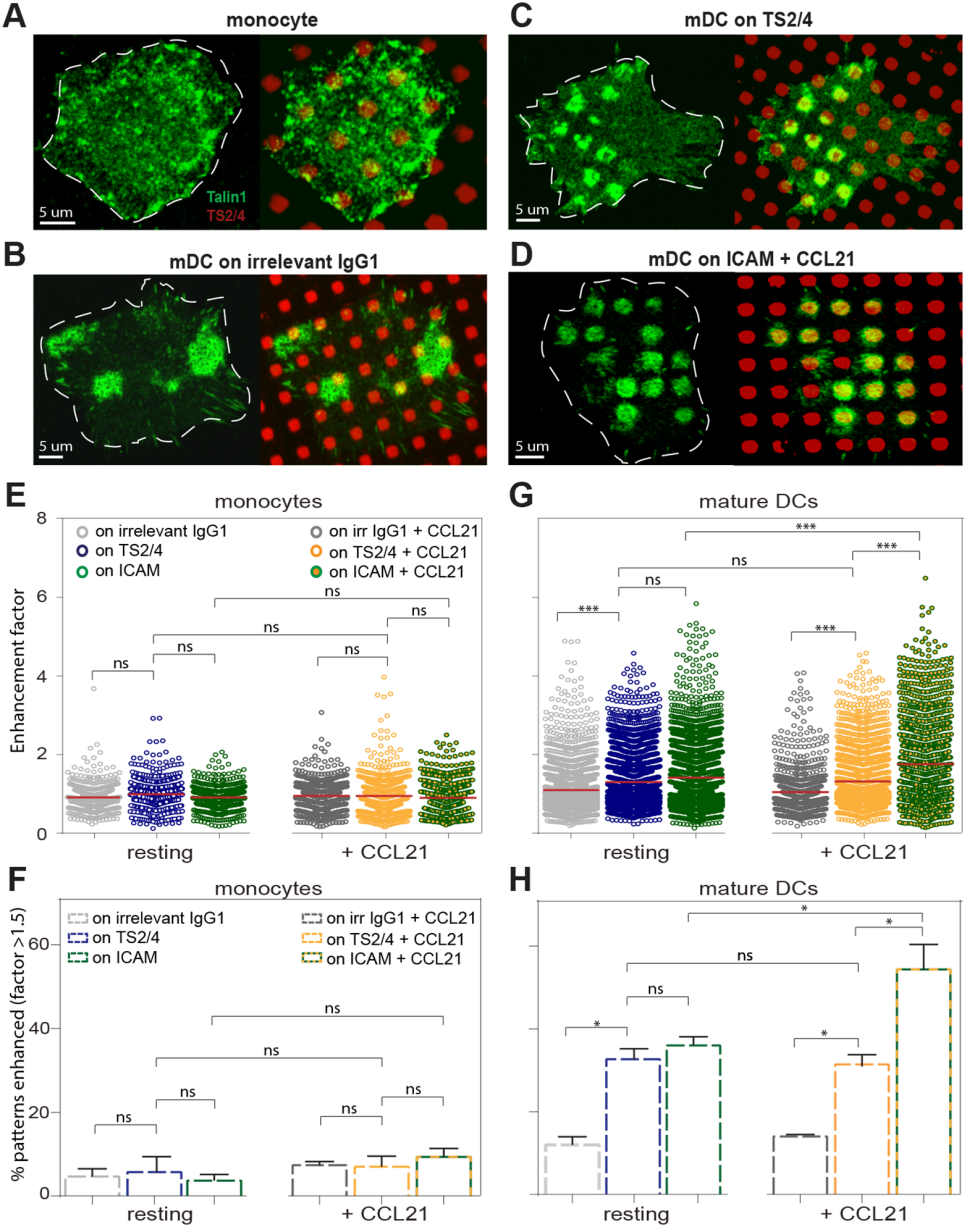
Localization of Talin1 to LFA-1 on monocytes and mDCs. (**A, B**) Representative images of (**A**) a monocyte seeded on a TS2/4 pattern, (**B**) a resting mDC on irrelevant IgG1 pattern, (**C**) a resting mDC seeded on a TS2/4 pattern and (**D**) a CCL21 activated mDC seeded on a ICAM-1 pattern. Green corresponds to Talin1 and red to the location of IgG1, TS2/4 or ICAM-1 positive squares. Cells are delineated by white lines. (**E**) Quantification of the degree of Talin1 enhancement to the positive areas in monocytes in different conditions (see Methods). Mean enhancement factor is displayed in red per condition. (**F**) Percentage of positive squares per experiment (n = 3, each a different donor) that showed significantly enhanced Talin1 signal per condition in monocytes. An enhancement factor of ≥1.5 was considered significantly enhanced, since 95% of the control sample (monocytes on IgG1) showed an enhancement factor below this value. (**G**) Quantification of the degree of Talin1 enhancement to the positive areas in mDCs in different conditions (see Methods). Mean enhancement factor is displayed in red per condition. (**H**) Percentage of positive squares per experiment (n = 3, each a different donor) that showed significantly enhanced Talin1 signal per condition in mDCs. Around 60 cells of 3 different donors were analyzed per condition. Monocytes contained 10 positive areas on average per cell, while mDCs contained around 50 positive areas. Means ± SEM are depicted. The Kruskal-Wallis test, followed by Dunn’s multiple comparison test was used to determine significant differences between means in E and G. The One-way ANOVA followed by the Tukey’s multiple comparison test were used to determine significant differences between means in F and H. The resulting P values are indicated as follows: *ns (*P>0.05); * (P<0.05) and *** (P<0.0001).

All analyses were performed using GraphPad Prism 6. Results are shown as the mean ± SEM. To determine statistical differences between the mean of two data sets (Figure 1), the (un)paired two-tailed Student T-test was used. To determine statistical differences between the mean of 3 or more data sets, the One-way ANOVA was used, followed by the Tukey’s multiple comparison test (Figures 2, 3 A–E, 4F and 4H). In the case of non-Gaussian distributed data sets (Figure 4E and 4G), statistical differences were calculated using the Kruskal-Wallis test, followed by Dunn’s multiple comparison test. Significance is represented using: *ns (*P>0.05); * (P<0.05); ** (P<0.001) and *** (P<0.0001).

## Results

### LFA-1 Mobility Increases During Differentiation of Monocytes into mDCs

We have previously shown on resting monocytes that lateral diffusion of LFA-1 across the membrane correlates with activation, with low-affinity LFA-1 being primarily mobile and high-affinity LFA-1 being immobile and anchored to the cytoskeleton [17], [36]. Moreover, we also showed that LFA-1 becomes inactive upon differentiation of monocytes into dendritic cells [15], [18]. Based on these results, we hypothesized that differences in LFA-1 activity between monocytes and mDCs might be also reflected in the lateral mobility of the receptor. We therefore recorded the diffusive behavior of LFA-1 on both cell types using previously established single particle tracking approaches [17]. We labeled LFA-1 under sub-labeling conditions using the conformation-independent antibody TS2/4 attached to the fluorophore ATTO647N on both primary monocytes and mDCs. We then recorded the mobility of individual diffusing fluorophores using a single molecule set-up working under oblique illumination, and subsequently reconstructed trajectories of the diffusing molecules. Whereas some molecules were highly mobile, other molecules showed much slower diffusion or were even stationary (Figure 1A, Movie S1). Individual trajectories were analyzed by generating mean square displacement (MSD) plots to obtain the diffusion coefficient (D) at short time lags. Histograms of the D values of all recovered trajectories on both cell types were created, displaying the full distribution of the diffusive behavior of LFA-1 (Figure 1B). A clear shift of the entire histogram towards higher D values is observed on mDCs compared to monocytes indicating an overall increase in lateral mobility of LFA-1 on mDCs. This increase on mobility was also accompanied by a modest but reproducible reduction on the stationary LFA-1 population (defined for values below 0.001 µm^2^/s) from 15% on monocytes to 10% on mDCs (Figure 1B, C).

To further enquire on the diffusive behavior of LFA-1 at longer observation times, we applied cumulative probability distribution (CPD) analysis [37]. This approach allowed us to separate the entire mobile LFA-1 population into two different fractions, namely a slow diffusing fraction and a fast diffusing fraction. We then calculated the percentage of molecules belonging to each fraction (stationary, slow or fast) (Figure 1C) and their respective average diffusion coefficients D (Figure 1D). Overall, mDCs exhibit a larger fraction of fast diffusing LFA-1 molecules at the expense of a smaller fraction of slow diffusing and stationary ones, compared to monocytes. Furthermore, the average diffusion coefficients of both the slow and the fast diffusing fractions of LFA-1 are considerably higher on mDCs than on monocytes. Altogether, these results show that the mobility of LFA-1 increases during differentiation of monocytes into mDCs and further suggests that the loss of ligand binding capacity of LFA-1 on mDCs might be correlated with its increased lateral mobility.

### Chemokine-induced LFA-1 Reactivation on mDCs Restricts LFA-1 Lateral Mobility

It is well established that chemokines trigger inside-out signaling events [38], [39] that lead to rapid LFA-1 activation and ICAM-1 mediated adhesion of lymphocytes [25] and mDCs [18]. We thus sought to investigate whether chemokine stimulation alters the diffusion profile of LFA-1 on mDCs. To this end, we reactivated LFA-1 on mDCs using CCL21 [18], [24], [25], a chemokine that regulates the homing of lymphocytes and dendritic cells from distant sites to lymphoid tissues [19], [20], and compared the diffusive behavior of the receptor before and after CCL21 activation. As CCL21 acts via binding to CCR7 [22], [23], we first confirmed the high expression level of this receptor on mDCs (Figure S1). Furthermore, because the activating effect of CCL21 on LFA-1 appears to be very rapid and transient within a few minutes [25], we carefully adjusted our single particle tracking experiments to measure LFA-1 mobility on mDCs 2 minutes after activation with CCL21, a time point at which the highest increase in LFA-1 dependent cell adhesion has been observed [25]. CCL21 stimulation significantly decreased LFA-1 mobility and increased the stationary population (Figure 2A) as compared to resting mDCs. Remarkably, the overall diffusion profile of chemokine-triggered re-activated LFA-1 on mDCs fully overlaps with that obtained on resting monocytes (Figure 2A). Indeed, a more detailed analysis shows that the slow and fast fractions (Figure 2B) as well as the diffusion coefficients thereof (Figure 2C) and stationary fractions (Figure 2D) between activated LFA-1 on mDCs and LFA-1 on resting monocytes entirely coincide.

To enquire whether the changes in LFA-1 mobility upon CCL21 activation are as transient as the activation of the receptor, we performed similar experiments at different time points after activation. The change in the percentages between stationary, slow and fast diffusing LFA-1 fractions (Figure 2E) as well as the change in average diffusion coefficients of the different fractions (Figure 2F) are indeed most prominent 2 minutes after activation. To fully verify that these results are indeed a consequence of CCL21 stimulation and not arising from experimental variations, we performed similar diffusion studies of LFA-1 at 1-minute intervals on mDCs without CCL21 stimulation (Figure S2 and S3). These controls, in which no significant differences are observed between indicated time points, confirm that the changes observed on mDC after CCL21 stimulation are real and maximal after 2 minutes.

After 4 minutes, changes due to CCL21 treatment are undetectable, indicating that the entire activation process is extremely transient. These results thus indicate that the activation state (i.e., ligand binding capacity) and mobility of LFA-1 are tightly and temporally correlated. Indeed, it appears that the loss of LFA-1 mediated cell adhesion during monocyte differentiation into mDCs correlates with an increased mobility of the receptor across the membrane. Transiently restoring LFA-1 adhesion function by conformational activation results in an equally transient decrease of mobility. In apparent contrast to our results, Constantin *et al*. have suggested that chemokine stimulation should trigger rapid integrin lateral mobility, which together with immediate triggering of the high-affinity state would cooperate in mediating rapid lymphocyte arrest under physiological conditions [25]. The apparent discrepancies between these findings and ours are discussed below.

### Binding of ICAM-1 Nano-aggregates, but not Monomeric ICAM-1, Magnifies Chemokine-induced Activation of LFA-1

Whether chemokines alone are sufficient to induce the high affinity state of LFA-1, or whether ligand binding is also necessary to fully bring the receptor in a stable high affinity conformation is not entirely clear. While some models suggest that chemokines alone induce the high affinity form of the integrin independent of ligand binding [25], others propose that chemokines trigger an extended form of low-to-intermediate affinity that is followed by a transition to a high affinity state upon interaction with the ligand [24]. More recently, it has been shown that affinity of LFA-1 for soluble monomeric ICAM-1 is only slightly increased upon LFA-1 priming by chemokines [40]. To shed some light into this controversy we performed single particle tracking experiments of LFA-1 in the presence of soluble monomeric ICAM-1, on resting and CCL21-stimulated mDCs. In addition, we also recorded the mobility of LFA-1 in the presence the ICAM-1 nano-aggregates (see Methods) to test the role of single *vs.* multi-ligand binding on the diffusion profile of the receptor.

We first measured the mobility of LFA-1 on resting, non-stimulated mDCs before and after adding soluble ICAM-1 monomers or nano-aggregates. Since LFA-1 on mDCs is mostly inactive and unable to bind the ligand, no changes in mobility are expected. Indeed, the diffusion profiles of LFA-1, i.e., stationary fraction and mobility (Figures 3A, B) before and after addition of ICAM-1 remained unchanged, confirming that neither soluble ICAM-1 monomers, nor nano-aggregates, significantly affect the mobility of LFA-1 on resting mDCs. These results also indicate that soluble ligands on their own (either monomeric or clustered) are not sufficient to trigger integrin activation.

We then measured LFA-1 mobility 2 minutes after adding together CCL21 and ICAM-1 to mDCs. Interestingly, addition of monomeric ICAM-1 did not affect LFA-1 lateral diffusion, while ICAM-1 nano-aggregates magnified the effect that CCL21 has on LFA-1 mobility (Figures 3B, D). Indeed, the LFA-1 stationary fraction increased significantly upon addition of ICAM-1 nano-aggregates in the presence of CCL21 (Figure 3C), while the diffusion coefficient of the fast fraction drops considerably (Figure 3D). Consistent with these results, confocal imaging of fluorescently labeled ICAM-1 (red) and the quantification of this fluorescent signal confirms that only the addition of ICAM-1 nano-aggregates in combination with CCL21 activation leads to significant ligand binding (Figure 3 E–I). The average baseline fluorescent signal per cell in resting and CCL21 activated cells with ICAM-1 monomers (which became dimers due to fluorescent antibody labeling) as well as in resting cells with ICAM-1 nano-aggregates is similar, while the signal is significantly higher in CCL21 activated cells with ICAM-1 aggregates (Figure 3E and Figures 3G–I). Counting the fluorescent spots confirms the increased binding of ICAM-1 nano-aggregates after CCL21 activation (Figure 3F and Figures 3H–I). Together, these data show once more the tight correlation between LFA-1 activation state and lateral mobility on the cell membrane, and importantly demonstrate that single/dimeric ligand binding in solution is not sufficient to stabilize the CCL21-triggered high affinity state of LFA-1 on mDCs, requiring for that receptor clustering via binding of multiple ligands.

### Talin1 is Involved in Basal LFA-1 Regulation on Resting mDCs but not on Monocytes

A major intracellular player known to contribute to integrin function regulation and activation is Talin1 [24], [27]–[30]. Talin1 is a cytoplasmic protein that mediates the link between LFA-1 and the cytoskeleton by binding the β_2_ subunit of LFA-1 to actin [32], [33]. Since we here observed major changes in the fraction of stationary, cytoskeleton bound LFA-1 molecules between monocytes and mDCs, and between mDCs before and after CCL21 activation and ligand binding, we next sought to investigate the involvement of Talin1 in this difference. We used microcontact printing to create square patterns of either TS2/4 binding Abs against LFA-1 or ligand ICAM-1 on glass coverslips following an established procedure [41]. As a control, we also used square patterns of mouse IgG1 isotype control antibodies not specific for LFA-1. We then seeded the cells on the patterned surfaces. TS2/4 or ICAM-1 on the glass binds to LFA-1 on the cell membrane and diffusion of the receptor results in accumulation of LFA-1 to the patterned regions. Talin1 was fluorescently labeled, and the accumulation of the fluorescent signal to the LFA-1 rich areas was quantified (Figure 4). In resting as well as CCL21 stimulated monocytes, a rather diffused distribution of Talin1 was observed (Figure 4A), independently on whether monocytes were seeded on a pattern of IgG1, TS2/4 or ICAM-1 (Figure 4E,F). This indicates that, in our experimental conditions, Talin1 in monocytes does not preferentially localize to LFA-1, neither in resting state nor upon ligand binding.

In marked contrast, Talin1 preferentially localized to LFA-1 rich regions on mDCs, even without activation of LFA-1 by chemokine or ligand binding (Figure 4C–D, G, H). Notice however that we already observe a basal level of Talin1 accumulation (non-pattern related) close to the substrate that is aspecific for LFA-1 (Figure 4B). This patchy Talin1 accumulation might correspond to the formation of podosomes by mDCs [42], an adhesive structure in which β_1_ and β_3_ integrins as well as Talin1 are involved [43].

In resting mDCs where LFA-1 is specifically recruited to the TS2/4 positive squares (Figure 4C), Talin1 accumulation to the patterns was significantly higher than to control IgG1 patterns (Figure 4G, H). Priming of LFA-1 captured by TS2/4 using CCL21 does not significantly increase Talin-1 accumulation beyond that observed in the resting state (Figure 4G, H). These results thus show a basal association of LFA-1 with Talin1 already on resting mDCs, which is not affected by transient chemokine activation of LFA-1.

In resting mDCs seeded on patterns of ICAM-1, a basal Talin1 recruitment similar to that on TS2/4 is observed (Figure 4G, H). Since ICAM-1 is also a ligand for the *αMβ_2_* integrin involved in podosomes [43], we cannot exclude that the Talin1 recruitment we observed is only partially LFA-1 specific, especially considering that LFA-1 on resting mDCs does not bind ICAM-1 very efficiently [15], [18], [25]. On the other hand, the combination of both CCL21 priming and ligand binding highly increases Talin-1 accumulation to the patterns (Figure 4D, G) up to a level where around 50% of the patterns shows significant enrichment of Talin1 (Figure 4H). This increase is fully LFA-1 specific, since CCL21 does not activate other β_2_ integrins. These results thus show that the interaction of Talin1 with LFA-1 is highly increased upon binding of chemokine primed LFA-1 to ICAM-1, indicating subsequent anchoring to the cytoskeleton as a result of ligand binding.

## Discussion

We have previously shown that during *in vitro* differentiation of monocytes towards immature and mature DCs, LFA-1 remains expressed at similar levels, but that only monocytes express a subpopulation of primed and functional LFA-1 [15], [18]. Furthermore, one of us recently showed an important role for CCL21 in the regulation of DC adhesive behavior by modulating LFA-1′s conformation activation state [18]. Although lateral mobility of the receptor is known to contribute to avidity regulation impacting on LFA-1 adhesive properties [17], dynamic studies of LFA-1 on resting and activated mDCs have been lacking so far. In here we addressed the differential mobility of LFA-1 on mDCs and compared it to that observed on monocytes. By dynamically tracing individual LFA-1 molecules on both cell types we now show that LFA-1 diffusion is significantly faster on mDCs compared to monocytes. Reactivating LFA-1 on mDCs with chemokine CCL21 transiently slows down the diffusion of LFA-1 and increases the fraction of stationary molecules. Remarkably, after 2 minutes of CCL21 stimulation, the diffusion profiles of LFA-1 on mDCs and monocytes become remarkably similar. Since a similar transient reactivation of LFA-1 by CCL21 has been observed at the functional level, namely the binding of the receptor to its ligand ICAM-1 and the contribution to cell adhesion [25], our results establish a strong link between LFA-1 function and its lateral mobility on the cell membrane.

Our results are in full agreement with other recently observed correlations between reduced mobility and high activity of LFA-1. Indeed, Cairo *et al* demonstrated that the mobility of LFA-1 bound to multivalent ICAM-1 ligands is highly reduced [26]. Moreover, Rossier *et al* showed that the average diffusion coefficient of integrins inside focal adhesions (FAs) is significantly lower compared to the average coefficient outside FAs, where integrins do not actively contribute to adhesion [30]. Finally, we recently showed that upon activation of LFA-1 on monocytes (by Ca^2+^ removal, Mn^2+^ or activating antibodies) lateral diffusion becomes highly impaired [17]. Altogether, these results demonstrate that integrin immobilization correlates with integrin activation. In contrast, Constantin *et al* observed that transient stimulation of LFA-1 by CCL21 led to the active, high affinity conformation of the receptor, and the formation of micrometer size LFA-1 clusters. Based on these data the authors postulated that CCL21-induced activation of the receptor should be accompanied by a rapid increase in lateral mobility so that microclusters are quickly formed [25]. Our results however reflect a higher level of complexity on LFA-1 function regulation. In fact our data support a model by which activation of LFA-1 on mDCs by CCL21 results in a small subset of “instantaneously” immobilized molecules as a direct result of CCL21 binding to its receptor CCR7, with the large majority of the remaining LFA-1 molecules still diffusing across the membrane. This small fraction of stationary molecules would serve as anchoring points for other diffusing molecules to become arrested and/or reduce their diffusion, thereby facilitating clustering. These clusters support the creation of stable adhesion spots necessary for cell adhesion. This scenario is able to explain the microclustering observed by Constantin *et al*, and provides a rationale as to why the effect of CCL21 is not instantaneous but takes about two minutes to be fully reached. On monocytes on the contrary, we previously demonstrated the presence of a subpopulation of primed, stationary LFA-1 nanoclusters on resting cells, with mobile nanoclusters continuously contributing to cell adhesion [17], [36]. The strong and dynamic interplay between nanoclustering and mobility might constitute thus a primary mechanism that differentially regulates LFA-1 activation.

We furthermore investigated the effect of CCL21 in the presence of the LFA-1 ligand ICAM-1. Based on conformational data, it has been proposed that binding of LFA-1 to ICAM-1 induces and/or stabilizes its high-affinity conformation [44], [45], a process termed ligand-induced activation. In our experimental conditions this high-affinity conformation has been transiently induced by CCL21. We found that soluble monomeric ICAM-1 (in combination with CCL21 stimulation) is not sufficient to further activate LFA-1 or to stably bind to the receptor. Nano-aggregates of ICAM-1 on the other hand significantly magnify the effect that CCL21 has on LFA-1 mobility and readily bind to LFA-1. We therefore postulate that ligand induced activation of LFA-1 as reported in literature is actually caused by LFA-1 clustering promoted by the proximity of multiple ICAM-1 molecules, i.e., a consequence of avidity. This is in agreement with both the notion that ligand affinity of LFA-1 is barely increased by chemokine activation [40], and studies showing that multivalent soluble ICAM-1 or fixed ICAM-1 substrates at high densities readily lead to strong immobilization of the receptor and LFA-1 mediated cell adhesion [26], [41].

More recently, it has been postulated that after ICAM-1 binding, postligand binding events must occur in order to fully bring LFA-1 in the high affinity state [40]. Since monomeric soluble ligands appear to barely trigger the high affinity of LFA-1 while fixed ligands do, it has been hypothesized that traction forces resulting from the translational motion of the integrin with respect to its bound, fixed ligand might contribute to further extend the β_2_ subunit of the integrin leading to the stabilization of its high affinity state, i.e. post ligand binding effects. [40]. However, this hypothesis is difficult to conciliate with abundant recent evidence, including the results shown in here that demonstrate that ready-to-bind-integrins are stationary on the cell membrane. With no actual mobility between the ligand and the receptor, the existence of traction forces is hard to explain. In the presence of shear forces however, the force that is needed to reinforce ligand binding does not come from receptor mobility but from external factors [46], [47], and post-ligand binding events can therefore occur.

One of the major intracellular players known to contribute to LFA-1 function regulation is Talin1. Knock-down and mutation of Talin1 has been shown to cause loss of ligand binding capacity of several β_1_ and β_3_ integrins [29], [48], leading to a model explaining integrin activation as an event triggered by structural separation of the α and the β subunits due to Talin1 binding to the β-leg thereby inhibiting interaction with the α-leg [28]. In here we show that activation of LFA-1 by chemokine CCL21 does not increase Talin1 association to the integrin, unless ligand binding stabilizes this transient activation. This suggests that Talin1 recruitment to LFA-1 is a result of integrin activation rather than a trigger thereof.

We further show preliminary evidence indicating that Talin1 might also play a different and more complex role in the regulation of β_2_ integrins like LFA-1. We find that Talin1 already colocalizes with LFA-1 on mDCs in the resting state prior to integrin activation. This indicates that Talin1 in β_2_ integrins is not only involved in maintaining LFA-1 in the active state, but also somehow in regulating resting, inactive LFA-1. Others have also observed association of Talin1 to LFA-1 on resting cells. Sampath *et al* showed that β_2_-integrins on resting neutrophils, which belong together with DCs to the group of antigen presenting cells in which LFA-1 is inactive in resting state, co-immunoprecipitates with Talin1 (225 kDa) [49]. Upon activation, β_2_ co-immunoprecipitates with a smaller talin1 (190 kDa) that corresponds to the head domain. In addition, Kim *et al* showed that transfecting K562 cells with the Talin1 head domain increases LFA-1 affinity and ligand binding [44]. Although in our experiments we could not discriminate between the full Talin1 or its head domain, it is clear that Talin1 does not exclusively associate to LFA-1 upon activation, but could instead provide the receptor with the possibility of becoming activated by additional factors such as chemokines.

In summary, our results highlight the importance of lateral mobility of LFA-1 across the membrane on the regulation of integrin activation and its function as adhesion receptor. We further demonstrate that chemokines alone are not sufficient to trigger the high affinity state of the integrin based on the strict definition that affinity refers to the adhesion capacity of a single receptor to its ligand in solution. Instead, our results are consistent with the notion that ligand induced activation of LFA-1 is a consequence of avidity. Finally, we provide preliminary evidence for an additional subtler role of Talin1 in regulating LFA-1 activation state, namely by being the agent on which activators such as chemokines can react to, rather than being the activating agent itself. We thus identified LFA-1 mobility, ligand binding and Talin1 recruitment as important players in the tight regulation of the homing of DCs from distant sites to the lymphatic tissues by chemokine CCL21.

## Supporting Information

Figure S1 **Expression level of CCL21 receptor CCR7 on the membrane of mDCs.** Isotype specific control and CCR7 signal are displayed, as well as the MFI of the CCR7 signal. Histogram is a representative out of 4 experiments.
(TIF)

Figure S2 **Control experiment 4 minutes CCL21: percentage.** Control experiments showing the percentage of the stationary, slow and fast diffusing LFA-1 on mDCs without CCL21 stimulation, at different time points. 6 cells (around 1000 trajectories) were measured per time point. Means ± SEM are depicted. The One-way ANOVA followed by the Tukey multiple comparison test were used to determine significant differences between means. The resulting P values are indicated as follows: *ns (P*>0.05); * (P<0.05) and *** (P<0.0001).
(TIF)

Figure S3 **Control experiment 4 minutes CCL21: D.** Control experiments showing the D values for the total mobile, and slow and fast fractions of LFA-1 on mDCs without CCL21 stimulation, at different time points. 6 cells (around 1000 trajectories) were measured per time point. Means ± SEM are depicted. The One-way ANOVA followed by the Tukey multiple comparison test were used to determine significant differences between means. The resulting P values are indicated as follows: *ns (P*>0.05); * (P<0.05) and *** (P<0.0001).
(TIF)

Movie S1 **Single LFA-1 mobility on mDCs.** Representative movie of TS2/4-ATTO647N labeled LFA-1 on a mDC to which SDT was applied. Individual fluorescent spots correspond to diffusive or stationary LFA-1 molecules. The background at the centre of the movie corresponds to the nucleus. Image area: 39×33 µm^2^ Frame rate: 10 Hz. Length of movie: 200 frames.
(AVI)
