# Supplementary figures and images for: Priming by Chemokines Restricts Lateral Mobility of the Adhesion Receptor LFA-1 and Restores Adhesion to ICAM-1 Nano-Aggregates on Human Mature Dendritic Cells
Source: PLoS One. 2014 Jun 19;9(6):e99589. doi: 10.1371/journal.pone.0099589 (PMC4063950; doi:10.1371/journal.pone.0099589)

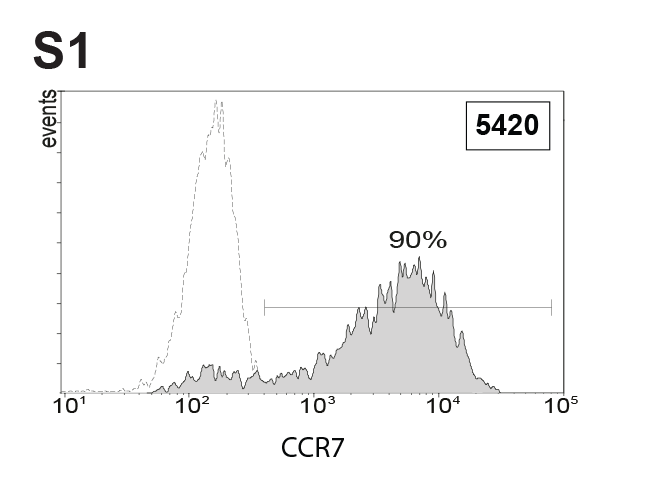

Supplement: Figure S1 — Expression level of CCL21 receptor CCR7 on the membrane of mDCs. Isotype specific control and CCR7 signal are displayed, as well as the MFI of the CCR7 signal. Histogram is a representative out of 4 experiments. (TIF) [file pone.0099589.s001.tif]

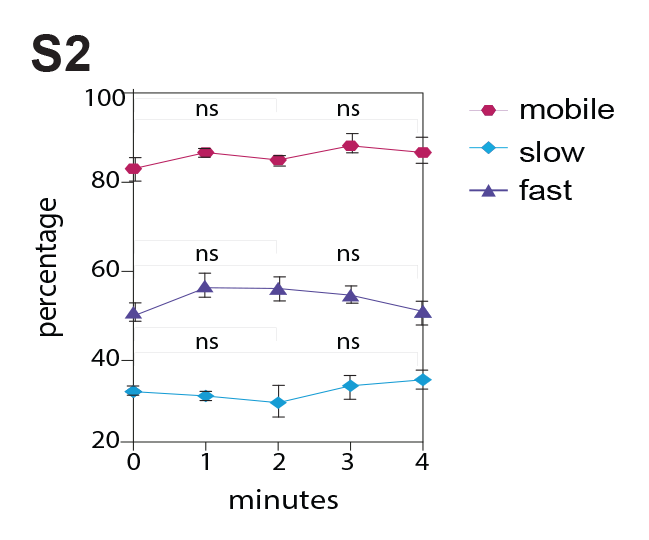

Supplement: Figure S2 — Control experiment 4 minutes CCL21: percentage. Control experiments showing the percentage of the stationary, slow and fast diffusing LFA-1 on mDCs without CCL21 stimulation, at different time points. 6 cells (around 1000 trajectories) were measured per time point. Means ± SEM are depicted. The One-way ANOVA followed by the Tukey multiple comparison test were used to determine significant differences between means. The resulting P values are indicated as follows: ns (P>0.05); * (P<0.05) and *** (P<0.0001). (TIF) [file pone.0099589.s002.tif]

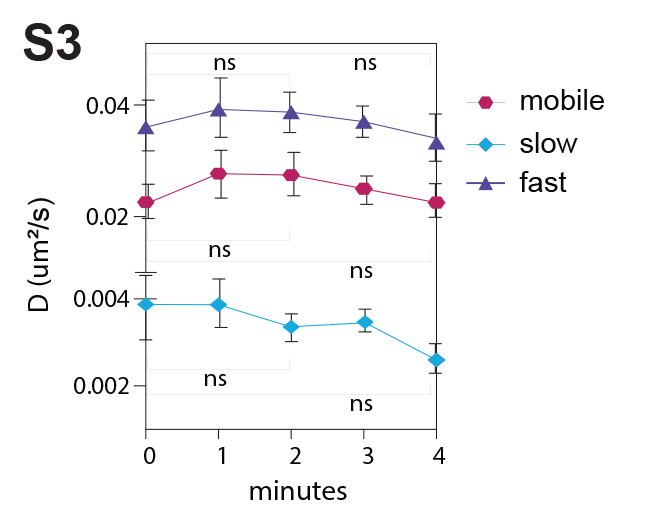

Supplement: Figure S3 — Control experiment 4 minutes CCL21: D. Control experiments showing the D values for the total mobile, and slow and fast fractions of LFA-1 on mDCs without CCL21 stimulation, at different time points. 6 cells (around 1000 trajectories) were measured per time point. Means ± SEM are depicted. The One-way ANOVA followed by the Tukey multiple comparison test were used to determine significant differences between means. The resulting P values are indicated as follows: ns (P>0.05); * (P<0.05) and *** (P<0.0001). (TIF) [file pone.0099589.s003.tif]
